# The clinical effectiveness of a self-management intervention for patients with persistent depressive disorder and their partners/caregivers: results from a multicenter, pragmatic randomized controlled trial

**DOI:** 10.1186/s13063-024-08033-9

**Published:** 2024-03-13

**Authors:** Ericka C. Solis, Ingrid V. E. Carlier, Noëlle G. A. Kamminga, Erik J. Giltay, Albert M. van Hemert

**Affiliations:** 1 Department of Psychiatry, Leiden University Medical Center, P.O. Box 9600, Leiden, 2300 RC The Netherlands; 2 Department of Psychiatry and Medical Psychology, Maastricht University Medical Center, Maastricht, The Netherlands; 3 Faculty of Health, Medicine and Life Sciences (MHeNs), Maastricht University, Maastricht, The Netherlands

**Keywords:** Clinical effectiveness, Randomized controlled trial, Self-management, Treatment resistant depression, Persistent depressive disorder, Psychiatric rehabilitation

## Abstract

**Background:**

Persistent depressive disorder (PDD) is prevalent and debilitating. For patients with PDD, psychiatric rehabilitation using self-management interventions is advised as the next therapeutic step after multiple unsuccessful treatment attempts. The “*Patient and Partner Education Program for All Chronic Diseases*” (PPEP4All) is a brief, structured self-management program that focuses on functional recovery for patients and their partners/caregivers. In chronic somatic disorder populations, PPEP4All has already been shown to be clinically effective. We examined whether PPEP4All adapted for PDD (PPEP4All-PDD, nine weekly group or individual sessions) is also clinically effective for adults/elderly with PDD and their partners/caregivers compared to care-as-usual (CAU) in specialized mental healthcare.

**Methods:**

In this mixed-method multicenter pragmatic randomized controlled trial, 70 patients with PDD and 14 partners/caregivers were allocated to either PPEP4All-PDD (patients, *n* = 37; partners/caregivers, *n* = 14) or CAU (patients*, n* = 33; partners/caregivers, not included) and completed questionnaires at 0, 3, 6, and 12 months regarding depressive symptoms, psychopathology, psychosocial burden, mental resilience, and happiness/well-being. Qualitative data were collected regarding treatment satisfaction. Data were analyzed using mixed model analyses and an intention-to-treat (ITT) approach.

**Results:**

There was no statistically significant difference in any outcome regarding clinical effectiveness between PPEP4All-PDD and CAU. Subgroup analysis for depressive symptoms did not show any interaction effect for any subgroup. Although 78% of participants recommended PPEP4All-PDD, there was no difference in treatment satisfaction between PPEP4All-PDD (score = 6.6; SD = 1.7) and CAU (score = 7.6; SD = 1.2), *p* = 0.06.

**Conclusion:**

Although depressive symptoms did not improve relative to CAU, this only confirmed that treatment for patients with treatment-resistant PDD should move from symptom reduction to functional recovery. Also, functional recovery may be reflected in other outcomes than psychosocial burden, such as self-empowerment, in patients with treatment-resistant PDD. Future research on PPEP4All-PDD could focus on a longer-term program and/or online program that may also be offered earlier in the treatment process as an empowerment intervention.

**Trial registration:**

Netherlands Trial Register Identifier NL5818. Registered on 20 July 2016 https://clinicaltrialregister.nl/nl/trial/20302

**Supplementary Information:**

The online version contains supplementary material available at 10.1186/s13063-024-08033-9.

## Introduction

Persistent depressive disorder (PDD) is a prevalent and debilitating psychiatric diagnosis that refers to suffering from depressive symptoms for a prolonged period of at least 2 years [1–3]. Compared to non-chronic/episodic depression, patients with PDD have a poor prognosis due to the persistence of depressive symptoms, reflected in poorer response to psychotherapy and antidepressants in specialized mental healthcare [4–8]; increased mental healthcare utilization [9]; and more frequent psychiatric hospitalizations [10, 11]. More intensive prolonged care (i.e., higher number of attempted psychotherapeutic or pharmacotherapeutic treatments) does not always mean better outcomes for patients with PDD: often they remain symptomatic for a longer time or experience more frequent recurrence of chronic episodes [4, 9, 12–15]. Even when depressive symptoms improve, patients with PDD may continue to experience residual social and occupational functional impairments and have difficulty finding meaning and enjoyment in life [16–18]. This leaves both clinicians and patients feeling powerless and frustrated [19]. These findings suggest an urgent need for psychiatric rehabilitation programs for PDD emphasizing functional recovery rather than symptom recovery, with greater focus on improving health-related quality of life [4, 20].

In the multidisciplinary depression treatment guidelines [21–23], psychiatric rehabilitation using self-management interventions is advised as the next therapeutic step for patients with multiple unsuccessful treatment attempts. However, in specialized mental healthcare, current regular care for patients with PDD often entails long-term, low-frequency, non-specific sessions, alongside maintenance pharmacotherapy [9, 24]. The “*Patient and Partner Education Program for All Chronic Diseases*” (PPEP4All) is a brief, specific protocolized program consisting of eight weekly group sessions that could meet this urgent need for a psychiatric rehabilitation program in mental healthcare. This PPEP4All program was previously evaluated in seven European countries (*EduPark-project*), and it has been shown to be clinically effective in patients with chronic physical disorders with or without comorbid depression and anxiety [25–32]. Crucial to our study, PPEP4All reduced depression scores on the Hospital Anxiety and Depression Scale (HADS) in groups of patients with various chronic somatic disorders and comorbid depressive symptoms [28]. This study suggests that a self-management program like PPEP4All could potentially reduce depression scores. Finally, research on PPEP4All has shown that involving the partner/caregiver may enhance treatment outcome of the patient and reduce the partner’s psychosocial burden concerning the patient’s disease [29–31, 33, 34]. However, current regular specialized mental healthcare usually does not include the partner or caregiver.

The current study evaluated the clinical effectiveness of PPEP4All adapted for PDD (PPEP4All-PDD), compared to care-as-usual (CAU), in adult and elderly patients with treatment-resistant PDD in specialized mental healthcare. In accordance with the PEPP4All (-PDD) protocol, partners/caregivers of PPEP4All-PDD patients were included. Similar to previous PPEP4All studies, we expected that completion of PPEP4All-PDD, compared to CAU, would lead to a greater decline in psychiatric symptom severity. With focus on functional recovery, we also expected more mental resilience and a greater sense of well-being in patients. Also, we expected that PPEP4All-PDD would result in lower psychosocial burden from chronic depression for both patients and their partner/caregiver.

## Methods

### Study design

The study was a mixed-methods, multicenter, pragmatic randomized controlled trial (RCT) that compared PPEP4All-PDD to CAU in patients with PDD. The pragmatic randomized controlled trial was selected to investigate PPEP4All-PDD in a way that mimics the real-world experience of the participating mental health clinics (e.g., the intervention was provided by trained mental health professionals of the clinic and inclusion criteria reflected the usual patient with PDD in the clinic). The primary focus of the study was the cost-effectiveness and quality of life/functioning of PPEP4All-PDD for patients with PDD (reported in a separate article); sample size was determined for this outcome. The secondary focus was the clinical effectiveness of PPEP4All-PDD in patients with PDD and comorbid psychiatric or somatic complaints (current article). The detailed methodology and design of this study have been reported elsewhere and is summarized below [35]. The study was approved by the Medical Ethical Committee (MEC) of the Dutch Leiden University Medical Center (LUMC).

Between April 2017 and March 2021, eligible patients were recruited from 11 locations of 5 Dutch mental healthcare organizations (see “Acknowledgements”) that offer secondary/specialized outpatient treatment for chronic depressive disorders. The main research center of this study was the Department of Psychiatry of the LUMC. Data collection was concluded in March 2022 in agreement with the financial sponsor of this project. Due to the low risk of harm of PPEP4All-PDD, as assessed by the MEC, interim analyses of clinical outcomes which guide potential stopping procedures were not deemed necessary. Our study and results were reported in accordance with CONSORT guidelines [36].

Patients included were adults/elderly (> 18 years) with PDD, and a treatment indication for psychiatric rehabilitation, as confirmed by the treating clinician. Exclusion criteria were severe psychopathology (e.g., schizophrenia, current psychotic state, severe substance addiction, bipolar disorder type I); acute and severe suicide risk; severe disabling somatic disorders; severe cognitive problems (e.g., dementia); expected medication changes during PPEP4All-PDD; currently receiving active psychotherapy; and insufficient Dutch fluency. Psychiatric nurses or psychiatrists of the participating mental healthcare locations identified patients eligible for participation and introduced research participation. Eligible patients were then formally screened by a trained research assistant for DSM-IV chronic depressive disorders with the Dutch translation of the Mini-International Neuropsychiatric Interview (MINI interview, modules A, B, and C regarding depression, dysthymia, and suicidality), which is a well-validated diagnostic interview used to identify psychiatric disorders, with excellent interrater and test–retest reliability [37, 38]. Severity of comorbid psychiatric or physical diagnoses were verified in relation to exclusion criteria; however, these data were not formally collected. After receipt of written informed consent, eligible patients were randomly allocated to either PPEP4All-PDD or CAU by an independent data coordinator, using a randomization schedule stratified by gender and mental healthcare location. The schedule was designed by an independent statistician of LUMC. For the patients allocated to PPEP4All-PDD, partners or caregivers (e.g., close family member, or close friend) were approached for participation in the project, after the patient agreed to inviting him/her. Prior to participation, we verified that partners/caregivers were not currently receiving active psychotherapy and were able to participate in PPEP4All-PDD partner sessions (minimum three sessions), due to pragmatic reasons.

Participants completed questionnaires using an online application, which is part of our Routine Outcome Monitoring (ROM) system (e.g., [39]), at the location of choice (i.e., home, mental health clinic, research center). Otherwise, research assistants could call the participant by appointment and complete the questionnaires with the participant. Additionally, on the signed consent form, participants indicated whether they agreed to be invited to a follow-up nested qualitative interview study in which we evaluated their satisfaction with PPEP4All-PDD. PPEP4All-PDD therapists were also invited to complete the qualitative interview, with a signed informed consent form.

### Measures

At the first assessment, all participants completed a demographic information questionnaire. Hereafter, at all assessments (i.e., 0, 3, 6, and 12 months), patients completed a test battery (see Table 1 of Supplementary Material for an overview of all questionnaires of the study). Partners/caregivers of PPEP4All-PDD patients only completed the psychosocial burden questionnaire. Partners/caregivers of CAU patients were not involved in CAU treatment and therefore did not participate in this study.

#### Depressive symptoms

For the clinical study, depressive symptoms were measured using the 28-item *Inventory of Depressive Symptomatology, Self-Report (IDS-SR)* [40–42]. Each item of the IDS-SR is scored on a 4-point Likert scale ranging from 0 to 3. A higher total score reflects higher depression severity, with total scores ranging from 0–84 (clinically-relevant score is ≥ 14) [40, 43]. The IDS-SR has shown adequate internal consistency (Cronbach’s alpha ranged from 0.92 to 0.94), test–retest reliability, as well as adequate convergent and discriminant validity [40].

#### General psychopathology

General psychopathology was measured using the 48-item *Symptom Questionnaire-48 (SQ-48)* [44–47]. Each item is rated on a 5-point Likert scale (0 = “Never”; 4 = “Very often”). We calculated total scores by summing the seven symptom subscales (social phobia, somatic complaints, depression, cognitive complaints, anxiety, agoraphobia, and hostility/aggression). A higher score reflects higher levels of psychopathology/symptoms, with total scores ranging from 0–184 (clinically relevant score is > 41) [44–47].

#### Mental resilience

Mental resilience was measured using the 6-item *Brief Resilience Scale (BRS)* [48, 49]. Each item is rated on a 5-point Likert scale (1 = “Strongly Disagree”; 5 = “Strongly Agree”). Items 2, 4, and 6 are reverse scored. The total scores (ranging from 6 to 30) are averaged by dividing by 6. Total scores reflect the level of mental resilience: 1.00 to 2.99 is “low”, 3.00 to 4.30 is “normal”, and 4.31 to 5.00 is “high” [49, 50].

#### Well-being/happiness

Well-being/happiness was measured using the 1-item *Self-Rated Happiness survey (SRH)* [51–53]. It is scored on a 7-point Likert scale (1 = “completely happy”, 2 = “very happy”; 6 “very unhappy”; 7 = “completely unhappy”). A higher score reflects lower levels of happiness/well-being [51].

#### Psychosocial burden

Psychosocial burden was measured in patients and partners/caregivers of PPEP4All-PDD patients using the Questionnaire on Burden of Chronic Disease for Patients (B4CZ) (based on the *Burden Questionnaire Parkinson Short Form, BELA-P-k* [54]) or the Questionnaire on Burden of Chronic Disease for Partners (B4CZ-Partner) (based on the *Burden Questionnaire for Relatives Short Form, BELA-A-k* [54]). The B4CZ and B4CZ-Partner were previously validated in Dutch [55, 56]. The B4CZ (19 items) and B4CZ-Partner (15 items) questionnaires provide two total scores regarding disease burden: bothered by (psychosocial) problems (“Bothered-by Problems”; *B4CZ-Bb, or B4CZ-Partner-Bb*) and perceived need for help (“Need for Help”; *B4CZ-NfH, or B4CZ-Partner-NfH*). Each item, scored on a 5-point Likert scale, explores the extent to which the patient or partner/caregiver is bothered by (Bb) the psychosocial problem (0 = “not at all” to 4 = “a great deal”), and the related need for some form of professional support/counseling concerning the psychosocial problem (NfH) (0 = “not important” to 4 = “very important”). Summing the items results in a total score, where higher scores indicate that the patient or partner/caregiver has higher levels of psychosocial burden and a greater need for help, respectively. For patients, scores from 20 to 38 are “moderate”, 39 to 57 are “high”, and 58 to 76 are “very high.” For partners/caregivers, scores from 16 to 30 are “moderate”, 31 to 45 are “high”, and 46 to 60 are “very high” [55, 57].

#### Treatment received (posttreatment)

For PPEP4All-PDD, we examined the number and format of sessions (based on attendance sheets) and whether patients changed medications during PPEP4All-PDD (based on a general evaluation survey). CAU patients were also asked on the evaluation survey regarding their treatment.

#### Treatment satisfaction (posttreatment)

Additionally, we evaluated satisfaction (rated on a scale of 1–10) with PPEP4All-PDD (among patients/caregivers/therapists) and CAU (patients). This included qualitative feedback from the evaluation survey and qualitative interviews (see interview topic list in Table 2 of Supplementary Material). In addition, we evaluated the percentage of patients who would recommend PPEP4All-PDD.

### Self-management intervention (PPEP4All-PDD, nine weekly sessions)

PPEP4All-PDD focuses on specific self-management themes, such as stress management, social skills building, and dealing with suicidality/crisis (see Figure A of Supplementary Material). Although the original PPEP4All program had eight sessions, it was previously suggested that an extra booster session after 3 months might help sustain enhanced quality of life over a longer period of time [31]. Taking this recommendation and the nature of PDD into account, PPEP4All-PDD included a ninth session focusing on suicidality, dealing with crises, and relapse prevention, and the fifth session included more information regarding chronic depression. The PPEP4All-PDD partner program took place separate from the patient program and involved the same themes as the PPEP4All-PDD patient program; however, the partner program was discussed from the partner/caregiver perspective.

In line with the original PPEP4All program, PPEP4All-PDD was primarily offered in group format. The group format was encouraged because participants could learn from each other, participants would have the opportunity to meet peers, and participants could gain (further) understanding and empathy from one another. However, in addition to the abovementioned adaptations, we made the following pragmatic modifications to PPEP4All-PDD, compared to the original PPEP4All program. First, to avoid unacceptably long waiting periods for participants, we allowed a minimum of 3 participants, instead of 5, to start groups. Second, to ensure that all participants could receive the intervention, one-on-one/individual sessions were also provided. This option was offered later considering that some participants preferred individual treatment or were unable to attend group sessions. Third, due to COVID-19 prevention regulations, we offered PPEP4All-PDD sessions also via online video calls. Finally, we allowed partners/caregivers to participate in a minimum of 3 sessions (out of 9), if they were unable to attend sessions (e.g., due to volunteering/work/childcare/illness).

During the intervention, PPEP4All-PDD patients and partners/caregivers received pharmacotherapy as required. In line with the recommendations of the original PPEP4All program, therapists and patients were requested not to change medication during PPEP4All-PDD [26]. Additionally, therapists were advised not to provide any additional psychotherapy sessions during PPEP4All-PDD. After completion of PPEP4All-PDD, patients could be referred to primary care or their general practitioner (GP). Otherwise, if necessary, patients could continue usual care sessions.

Each PPEP4All-PDD session was guided by a PPEP4All-PDD therapist who completed a 3-day certified course [29]. After the training, all PPEP4All-PDD therapists could receive continuous support from the PPEP4All-developer/founder (NK) (e.g., booster training session, intervision, national PPEP4All symposium). To ensure the quality of the program, each PPEP4All-PDD therapist worked with the PPEP4All-PDD handbook/protocol [58–60], which described each session in detail. After each session, the PPEP4All-PDD therapist completed a *Checklist of PPEP4All-PDD Protocol Treatment Integrity*, which included each component/theme listed in the PPEP4All-PDD handbook/protocol (video/sound recordings were not possible). This form was sent by the PPEP4All therapist to the coordinating researcher (ES) after completion of the final session of a patient or partner/caregiver group. Sufficient treatment protocol adherence (> 85%) was demonstrated in a random sample of 9 patient groups and 4 partner/caregiver groups, with the exception of the fifth partner/caregiver program, which ended after three sessions. The scoring of the checklists reflected excellent overall agreement between two independent raters (interclass correlation: 0.99, with 95% confidence interval, 0.99–1.00).

### Care as usual (CAU)

After two or more unsatisfactory treatment options, patients with CAU typically receive long-term, non-protocolized supportive care from a psychiatric nurse or psychologist/psychotherapist, with pharmacological maintenance therapy from a psychiatric nurse specialist or psychiatrist. CAU was not confined to a maximum number of sessions within a fixed time period, and therefore, it could potentially continue during the entire study period of 1 year. Additionally, CAU was generally continued with the same therapist, offered with an individual treatment format, and did not include the partner/caregiver of the patient.

### Data analysis

To compare differences between the groups on baseline demographics and pre-test clinical variables, we used *t*-tests for continuous variables and chi-square (*χ*) tests for categorical variables. To test whether participants with any missing data differed on the demographic and clinical variables, we first created a dummy variable pertaining to whether any questionnaire was missing at any assessment point. To test whether there was any difference between groups for number of drop-outs, we created another dummy variable for attrition. These were tested using chi-square (*χ*) tests for categorical variables.

The scores between PPEP4All-PDD and CAU groups on depressive symptoms (IDS-SR), psychopathology symptoms (SQ-48), mental resilience (BRS), well-being/happiness (SRH), bothered-by (psychosocial) problems (B4CZ-Bb), and need for help (B4CZ-NfH) were analyzed. Effects were tested with repeated measures linear mixed-models analysis using the restricted maximum likelihood (REML) estimation procedure and an unstructured covariance matrix. Although linear mixed-models analysis can handle data missing at random [61], we also used an intention-to-treat (ITT) analysis with the last observation carried forward (LOCF) to account more conservatively for incomplete data. Time was coded as 0 for T1 (baseline), 3 (months) for T2, 6 (months) for T3, and 12 (months) for T4 and was included as a categorical repeated factor. We included the effect of time, condition (PPEP4All-PDD versus CAU), and the interaction of time × condition. These analyses were examined using superiority testing. There were no binary clinical outcomes to report.

We conducted a subgroup analysis on the IDS-SR and tested the interaction term with group for gender, age (based on mean age), education level, living situation, anxiety comorbidity (based on mean subscale score on the SQ-48), and psychosocial burden (based on mean score on B4CZ-Bb and B4CZ-NfH). We could not explore the possible effect of treatment modality (group/individual) nor ethnicity or work due to insufficient number of patients (*n* < 10). Descriptive analyses were conducted in IBM SPSS version 25, and mixed-model and subgroup analyses were conducted using the “lme4” package in R statistical software (R version 4.1.1.; R Foundation for Statistical Computing, Vienna, Austria, 2016. URL: https://www.R-project.org/). Significance for all statistical tests was set at* p* < 0.05 (two-sided).

## Results

### Participant flow and descriptive statistics

**Fig. 1:**
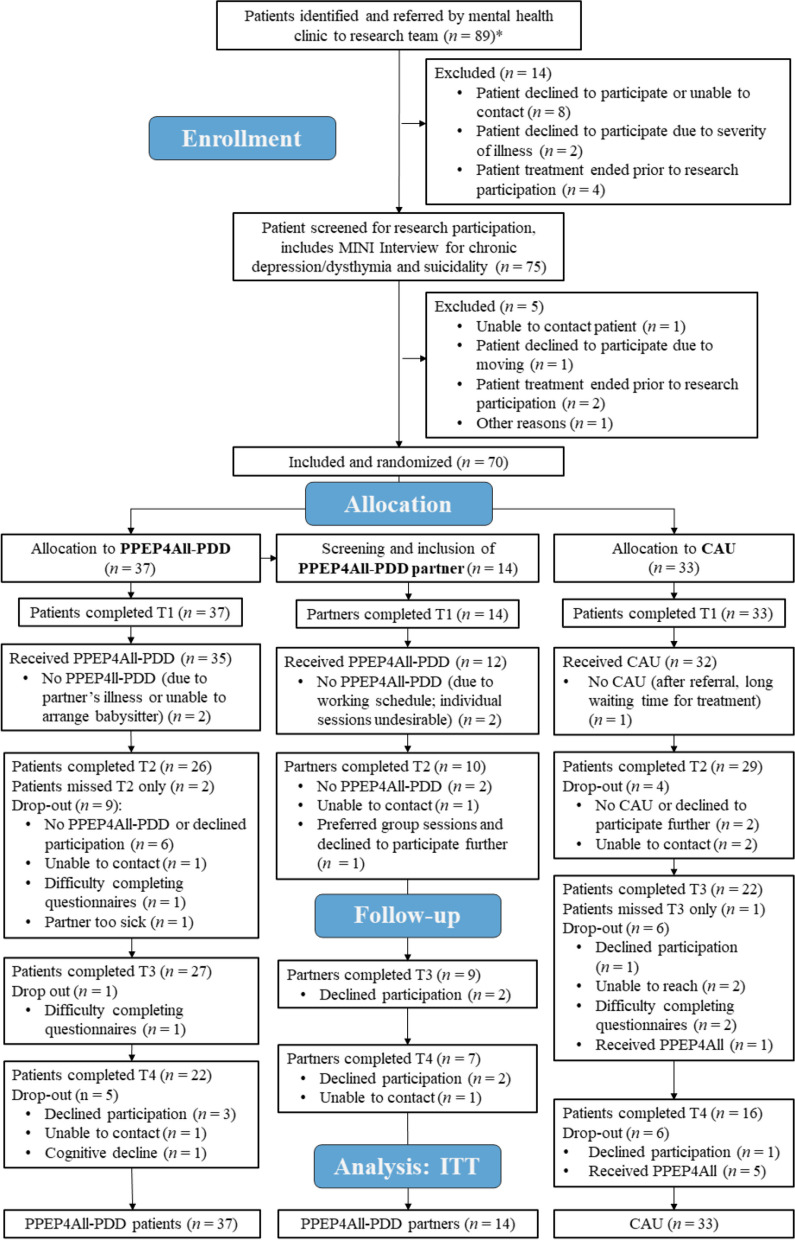
CONSORT flow diagram

Seventy patients with PDD were recruited and allocated to PPEP4All-PDD (*n* = 37) or CAU (*n* = 33). This included 14 PPEP4All-PDD partners/caregivers. Follow-up assessments were completed for all participants in March 2022. The participant flow is shown in the CONSORT Flow Diagram in Fig. 1.

The socio-demographic and clinical characteristics of the participants are shown in Table 1.

**Table 1:** Sociodemographic and clinical characteristics of PPEP4All-PDD and CAU participants

Characteristic	Total (*N* = 70)	PPEP4All-PDD (*n* = 37)	PPEP4All-PDD partner (*n* = 14)	CAU (*n* = 33)	Test statistic	*p*-value
Age, mean (SD)	57 (11.7)	55 (10.4)	58 (14.6)	59 (12.8)	*t*(68) = − 1.31	0.19
Gender						
Female, *n* (%)	48 (68.6%)	26 (70.3%)	6 (42.9%)	22 (66.7%)	*χ*^2^(1) = 0.10	0.75
Male, *n* (%)	22 (31.4%)	11 (29.7%)	8 (57.1%)	11 (33.3%)		
Ethnic background^a^						
Dutch, *n* (%)	53 (75.7%)	29 (78.4%)	12 (85.7%)	24 (72.7%)	*χ*^2^(1) = 0.30	0.58
Other, *n* (%)	17 (24.3%)	8 (21.6%)	2 (14.3%)	9 (27.3%)		
Education level^b^						
Lower education, *n* (%)	23 (32.9%)	26 (70.3%)	3 (21.4%)	21 (63.6%)	*χ*^2^(1) = 0.35	0.55
Higher education, *n* (%)	47 (67.1%)	11 (29.7%)	11 (78.6%)	12 (36.4%)		
Employment status^c^						
Employed, *n* (%)	7 (10.0%)	4 (10.8%)	5 (35.7%)	3 (9.1%)	*χ*^2^(1) = 0.06	1.00
Unemployed/retired, *n* (%)	63 (90.0%)	33 (89.2%)	9 (64.3%)	30 (90.9%)		
Marital status						
Married/cohabitating, *n* (%)	39 (55.7%)	21 (56.8%)	11 (78.6%)	18 (54.5%)	*χ*^2^(1) = 0.03	0.85
Other (single, widowed, divorced), *n* (%)	31 (44.3%)	16 (43.2%)	3 (21.4%)	15 (45.5%)		
Depressive symptoms (IDS-SR), mean (SD)	34.5 (14.9)	33.6 (15.3)	–	35.6 (14.6)	*t*(68) = − 0.54	0.59
Psychopathological symptoms (SQ-48), mean (SD)	62.5 (27.4)	61.73 (31.0)	–	63.33 (23.1)	t(68) = − 0.24	0.81
Bothered-By Problem (B4CZ-Bb), mean (SD)^d^	25.6 (15.9)	27.1 (16.0)	8.3 (8.5)	24.1 (15.8)	*t*(67) = 0.79	0.43
Need for help (B4CZ-NfH), mean (SD)	27.8 (16.2)	28.6 (17.9)	12.1 (11.0)	26.9 (14.3)	*t*(67) = 0.42	0.68
Mental resilience (BRS), mean (SD)	2.3 (0.7)	2.3 (0.7)	–	2.2 (0.7)	*t*(68) = 0.43	0.67
Well-being/unhappiness (SRH), median (IQR)^e^	4.5 (2.0)	5.0 (2.0)	–	4.0 (2.0)	*U* = 553.00	0.48

*PPEP4All-PDD* Patient and Partner Education Program for All Chronic Diseases-Persistent Depressive Disorder, *CAU* care as usual, *SD* standard deviation, *IDS-SR* Inventory of Depressive Symptomatology-Self Report, *SQ-48* Symptom-Questionnaire 48, *B4CZ* Questionnaire on Burden of Chronic Disease for Patients, *BRS* Brief Resilience Scale, *SRH* Self-Rated Happiness, *IQR* interquartile range

^a^Dutch ethnic background was assumed when the patient and both parents were born in the Netherlands

^b^Lower education was defined as having completed elementary school, lower general primary education or no education at all, whereas higher education was defined as having more than lower education and includes university studies

^c^Tested using Fisher’s exact probability test

^d^Bothered-By Problem also referred to Psychosocial Burden

^e^Tested using Mann–Whitney *U* test, and higher score reflects higher levels of unhappiness

Among the 70 patients (total ITT sample), 68.6% were female (48 out of 70). Most had completed higher education (67.1%), were married or cohabitating (55.7%), were unemployed or retired (90.0%), and from a Dutch ethnic background (75.7%). Patients were on average 57 (SD = 11.7, range 22–77) years old. When comparing PPEP4All-PDD and CAU patients, no differences were found in socio-demographic and clinical characteristics,* p* > 0.05. PPEP4All-PDD partners/caregivers were on average 58 years (SD = 14.6, range 26–74) and 42.9% were female.

There were 98.6%, 74.3%, 62.9%, and 52.9% outcome data available at T1, T2, T3, and T4, respectively. In total, there were approximately 26.4% missing values. We compared participants with and without missing data: participants with missing data had a higher percentage of lower education level, *χ*(1) = 5.5, *p* = 0.02. There was no significant difference in initial values on questionnaires or other sociodemographic characteristics between those with and without missing data*, p* > 0.05. There was also no statistically significant difference in condition (PPEP4All-PDD or CAU) for missing data, *χ*(1) = 0.03, *p* = 0.87, and for total number of drop-outs (*n*_ppep4all_ = 14; *n*_cau_ = 17), *χ*(1) = 1.32, *p* = 0.25. Examining drop-outs per time point was also not statistically significant, *χ*(3) = 7.36, *p* = 0.06.

### Treatment received

PPEP4All-PDD patients and their partners/caregivers completed, on average, 7 out of the 9 total sessions. Of the PPEP4All-PDD patients, 62.2% (*n* = 23) did not have a participating partner/caregiver in the PPEP4All-PDD partner program. Additionally, 89.2% (*n* = 33) participated in group format, compared to 11% (*n* = 4) in individual format. During the COVID-19 pandemic, 3 group sessions were provided by video calls. In total, there were 2 PPEP4All-PDD patients who did not start the program, and 8 dropped out early (< 6 sessions). Of the 14 PPEP4All-PDD partners, 2 dropped out early (< 3 sessions). There were 23 out of 37 (62.2%) PPEP4All-PDD patients and 10 out of 14 (71.4%) PPEP4All-PDD partners who completed a minimum of 6 PPEP4All-PDD sessions and who also completed at least the second assessment. Additionally, 19 of 37 (51.3%) PPEP4All-PDD patients and 4 out of 14 (28.6%) partners completed all 9 sessions. Considering the minimum for PPEP4All-PDD partners, 12 out of 14 (85.7%) PPEP4All-PDD partners completed at least 3 sessions.

We expected CAU patients to receive mainly supportive non-protocolized/unspecified sessions, which was only partially confirmed for the content of CAU: medication checks only (*n* = 2), supportive/unspecified sessions (*n* = 9), cognitive behavioral therapy (*n* = 5), mindfulness therapy (*n* = 1), and referral to another department (*n* = 1).

When examining medication changes, 12 out of 19 (66.7%) PPEP4All-PDD patients changed medication during the study, after completion of PPEP4All-PDD. Of the CAU patients, 4 out 20 (20.0%) changed medication during the study. In general, there were significantly more PPEP4All-PDD than CAU patients with any medication change during the study (*χ*(1) = 7.50, *p* = 0.01).

### Mixed model analyses

**Fig. 2:**
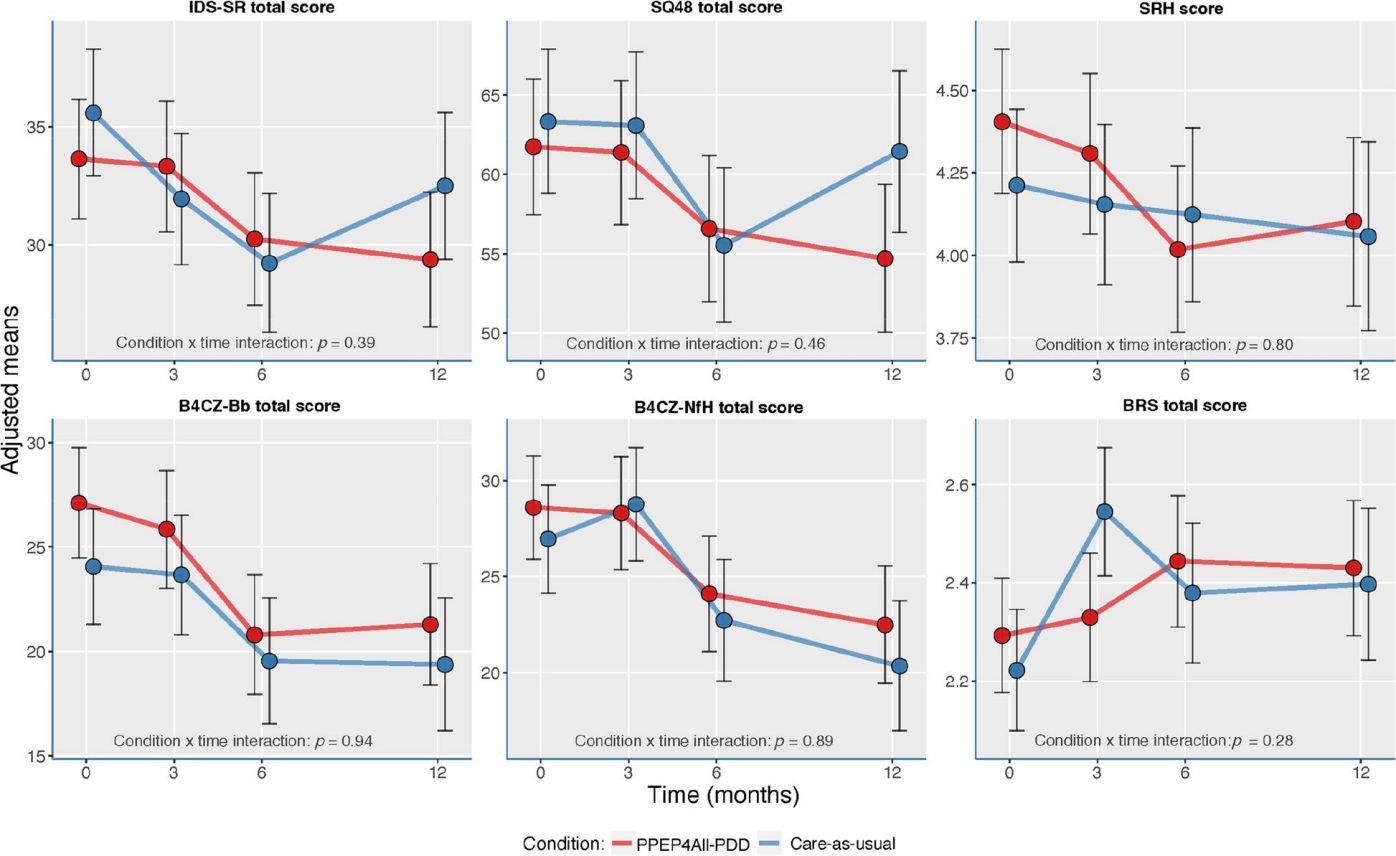
Mixed model analyses for the clinical effectiveness outcomes at each time assessment

First, we conducted repeated measures linear mixed-models analysis for patient outcome measures. Figure 2 shows the results of the mixed-model analyses with interaction effects with time at each time assessment. Table 3 in the Supplementary Material shows adjusted mean differences of the CAU and PPEP4All-PDD conditions per assessment point of the mixed-models. Depressive symptoms (IDS-SR) and psychiatric symptoms (SQ-48) decreased progressively between 0 months (T1) and 6 months (T3) for both groups. Between 6 months (T3) and 12 months (T4), depressive and psychiatric symptoms worsened (i.e., increased) for CAU patients, while symptom scores decreased for PPEP4All-PDD patients. The differences in adjusted means, however, for the IDS-SR and SQ-48 were not statistically significant at T3 nor T4 (see Supplementary Table 3). Well-being/happiness scores (SRH) did not greatly change for both groups across assessments despite subtle fluctuations in scores for PPEP4All-PDD patients between 3 months (T2) and 12 months (T4), compared to CAU patients (see also Supplementary Table 3). Regarding the B4CZ-Bb and B4CZ-NfH subscales, “psychosocial burden” and “need for help” scores decreased between 3 months (T2) and 6 months (T3) for both groups. Between 6 months (T3) and 12 months (T4), need for help decreased for both groups, while psychosocial burden remained unchanged for both groups, resulting in a similar level of improvement for both groups on both subscales. Finally, mental resilience (BRS) did not greatly change for both groups across assessments despite slightly higher scores for CAU patients, compared to PPEP4All-PDD patients, at the 3-month assessment (T2) (see also Supplementary Table 3). In general, all main effects (not shown in Fig. 2) and the condition × time interaction effects for all outcome measures were not statistically significant, *p* > 0.05. Thus, despite some (non-significant) differences between conditions in the trajectory of the outcome scores over time, broader examination of these scores revealed that, overall, there were no significant differences in outcomes between CAU and PPEP4All-PDD. Explorative mixed-model analysis for patient outcome measures, adjusted for medication changes, resulted in no statistically significant main effects nor condition × time interaction effects,* p* > 0.05 (see Supplementary Material).

**Fig. 3:**
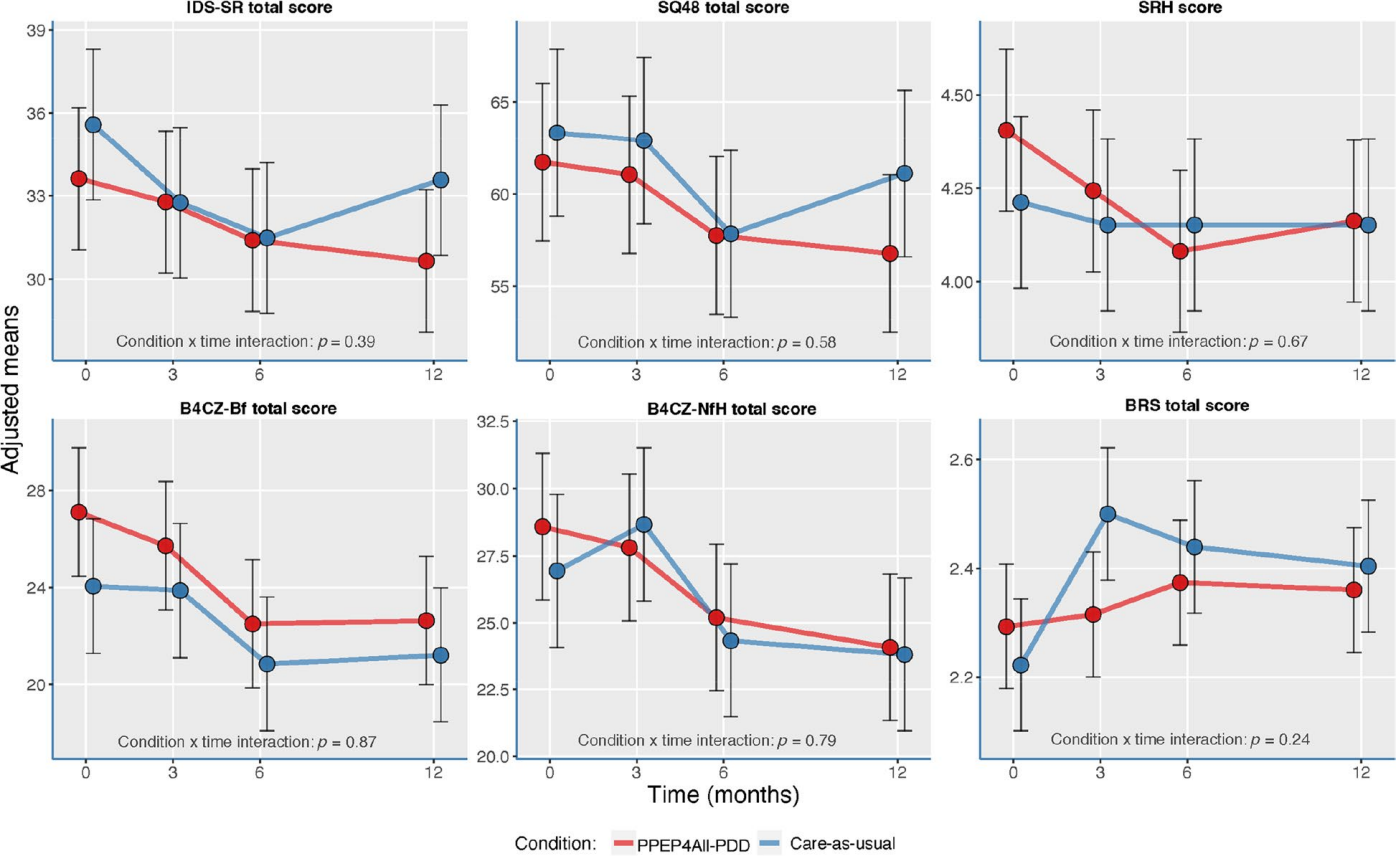
Intention-to-treat analysis using last observation carried forward (ITT-LOCF) for the clinical effectiveness outcomes at each time assessment

Figure 3 shows the results for the ITT-LOCF mixed-model analyses. In comparison with the previous mixed-model analyses, the ITT-LOCF analyses showed similar trends in depressive symptoms, psychiatric symptoms, well-being/happiness, psychosocial burden, need for help, and mental resilience. Similarly, there were (minor) improvements between 0 months (T1) and 12 months (T4) assessments in outcomes for both CAU and PPEP4All-PDD patients, with some (non-significant) fluctuations in outcome scores. Of note, depression symptoms (IDS-SR) and psychiatric symptoms (SQ-48) increased between 6 months (T3) and 12 months (T4) for CAU patients, while scores slightly decreased for PPEP4All-PDD patients. Well-being/happiness scores (SRH) did not greatly change, despite showing minor fluctuations for PPEP4All-PDD patients between 3 months (T2) and 12 months (T4) compared to CAU patients. Psychosocial burden (B4CZ-Bb) and need for help (B4CZ-NfH) changed similarly for CAU and PPEP4All-PDD patients across assessments, showing some improvement across assessments for both groups. Mental resilience (BRS) did not greatly change, despite slightly higher scores for CAU patients at 3 months (T2) than PPEP4All-PDD patients. Despite these fluctuations, all main effects (not shown) and condition × time interaction effects for all outcome measures remained statistically nonsignificant, *p* > 0.05.

**Fig. 4:**
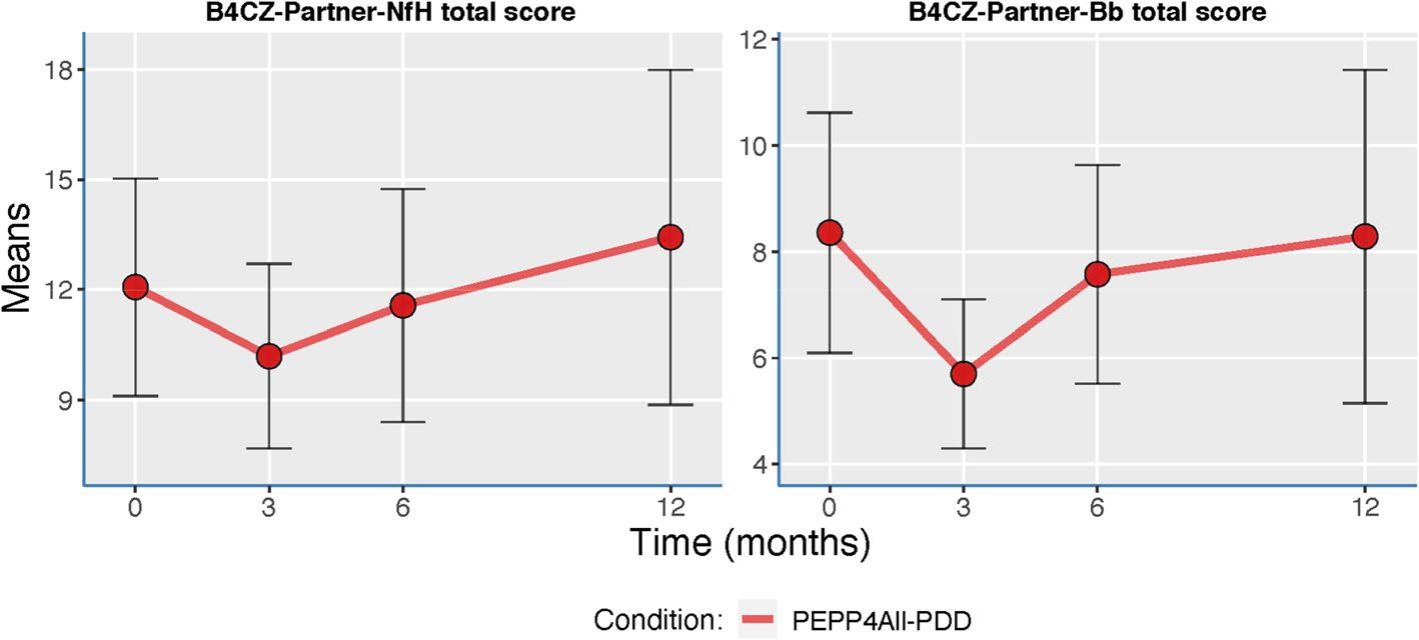
Psychosocial burden (B4CZ-Partner-Bb) and need for help (B4CZ-Partner-NfH) across the assessment points*.* B4CZ-Partner Questionnaire on Burden of Chronic Disease for Partners

For the PPEP4All-PDD partners, we examined the total scores on the B4CZ-Partner using related-samples Wilcoxon rank test, due to nonnormality of scores (see Fig. 4).

Figure 4 shows the caregiver/partner psychosocial burden (B4CZ-Partner-Bb) and need for help (B4CZ-Partner-NfH) across the assessment points. There was no significant difference in psychosocial burden at 0 (T1; *pre-PPEP4All-PDD*) and 3 months (T2; *post-PPEP4All-PDD*), *p* = 0.09, and no significant difference in psychosocial burden between 0 (T1; *pre-PPEP4All-PDD*) and 12 months (T4; *1-year follow-up*), *p* = 0.92. Similarly, there was no significant difference in need for help between 0 (T1; *pre-PPEP4All-PDD*) and 3 months (T2; *post-PPEP4All-PDD*), *p* = 0.31, and no significant difference in need for help between 0 (T1; *pre-PPEP4All-PDD*) and 12 months (T4; *1-year follow-up*), *p* = 1.00.

### Subgroup analyses

**Fig. 5:**
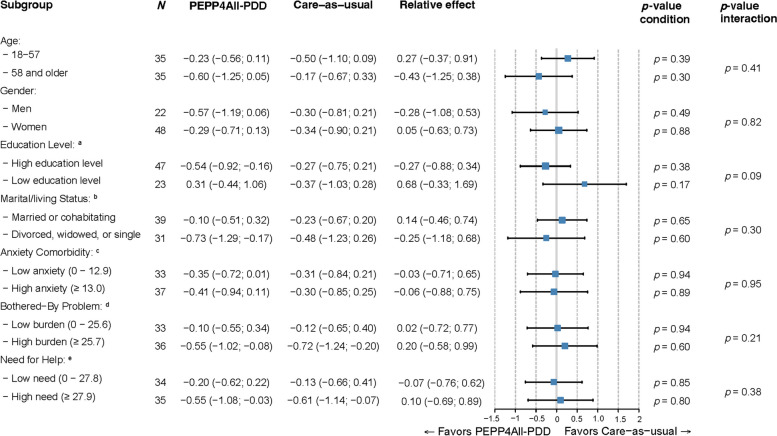
Subgroup analysis for depressive symptoms using the Inventory of Depressive Symptomatology. ^a^Lower education was defined as having completed elementary school, lower general primary education or no education at all, whereas higher education was defined as having more than lower education and includes university studies. ^b^Married/cohabitating were combined; single, widowed, divorced were combined. ^c^Based on the anxiety subscale of the Symptom-Questionnaire 48 (SQ-48), and divided into two groups using the total group mean. ^d^Based on the Bothered-by Problem total score of the Questionnaire on Burden of Chronic Disease for Patients (B4CZ-Bb), and divided into two groups using the total group mean. Referred to as Psychosocial Burden. ^e^Based on the Need for Help total score of the Questionnaire on Burden of Chronic Disease for Patients (B4CZ-NfH), and divided into two groups using the total group mean

Figure 5 shows a forest plot of PPEP4All-PDD versus CAU for the different subgroups. When looking at PPEP4All-PDD and CAU for gender, age, marital/living status, anxiety comorbidity level, psychosocial burden, and need for help, we noted that IDS-SR scores decreased. These main effects, however, were not statistically different among the groups, *p* > 0.05, and the interaction for the subgroup × condition × time was not statistically significant for the aforementioned subgroups, *p* > 0.05. The education level subgroup showed a notable pattern: for those with higher education, depression symptoms improved over time for both conditions; and for those with lower education, depression symptoms worsened for PPEP4All-PDD but improved for CAU at 1-year follow-up. The interaction effect for education level, however, was not statistically significant, *p* = 0.09.

### Treatment satisfaction

Patients rated the PPEP4All-PDD (*n* = 27) an average score of 6.6 (SD = 1.7) out of 10 and CAU (*n* = 14) an average of 7.6 (*SD* = 1.2) out of 10; the means were not statistically significant, *t*(39) = 1.92, *p* = 0.06. Of the PPEP4All-PDD patients who completed the final evaluation survey (*n* = 18), 77.8% (*n* = 14) patients recommended the PPEP4All program. PPEP4All-PDD partners/caregivers (*n* = 9) scored PPEP4All-PDD an average of 7.8 (SD = 0.6). PPEP4All-PDD therapists (*n* = 10) scored PPEP4All-PDD an average of 7.9 (SD = 0.5).

### PPEP4All-PDD treatment feedback

PPEP4All-PDD patients/caregivers/therapists all provided qualitative feedback regarding the program. This feedback was divided into “compliments” and “suggestions” (see Table 4 of Supplementary Material).

Overall, *compliments* about the PEPP4All-PDD program mainly touched on the following aspects: (1) group and individual format (specific advantages/choice); (2) skills learned; (3) workbook availability; (4) program goal/organization/themes; (5) caregiver involvement; (6) positive therapist aspects; and (7) patients’ positive posttreatment changes (e.g., psychosocial and motivational changes; mentioned by patients/caregivers).

*Suggestions* for improvement mainly concerned: (1) biweekly sessions (instead of weekly); (2) follow-up session(s) or program extension; (3) clearer rules regarding absences/drop-outs; (4) clarifying partner/caregiver's expectations prior to program; (5) therapist providing more examples to clarify workbook/homework; (6) workbook revision (i.e., shorter, more PDD-specific); (7) digital app use for homework/self-evaluation; (8) group program with two therapists (instead of just one); (9) maintaining small groups (max. 6); and (10) offering PPEP4All-PDD program (also) earlier in depression treatment process.

## Discussion

### Main findings

Our aim was to examine the clinical effectiveness after 1-year of the PDD-adapted “*Patient and Partner Education Program for All Chronic Diseases-Persistent Depressive Disorder”* (PPEP4All-PDD), compared to care-as-usual (CAU), in patients with PDD and their partners/caregivers in specialized mental healthcare, in a randomized controlled trial. Contrary to our hypotheses, we found no evidence for the superiority of PPEP4All-PDD compared to CAU in terms of clinical effectiveness. Although 78% of patients recommended PPEP4All-PDD, there was no difference in treatment satisfaction between PPEP4All-PDD and CAU. Our qualitative data regarding treatment satisfaction (based on an evaluation survey and individual qualitative interview) revealed potential improvements for PPEP4All-PDD (see Supplementary Material).

### Comparison to other studies

Previous studies have shown the clinical effectiveness of the original/generic PPEP4All program for patients with chronic somatic diseases, such as Parkinson’s disease [29, 31]; symptom-manifested Huntington’s disease [27]; and chronic pituitary disorders [32]. PPEP4All also reduced depression scores on the Hospital Anxiety and Depression Scale (HADS) in a non-controlled study in a sample of patients with various chronic somatic disorders and depressive symptoms [28]. In our trial, however, we found no evidence for the effectiveness of PPEP4All-PDD on depression scores nor other secondary clinical outcomes. Our trial differed from the aforementioned studies in terms of patient population, research/treatment design (e.g., PPEP4All-PDD adaptations), and outcome measures, which may explain the difference in results.

Results have been inconsistent across RCT studies investigating self-management programs for depression. Some programs resulted in improved depression severity and quality-of-life/self-efficacy outcomes for persons with mild depression (e.g., *COPERS, Pacifica, iFightDepression)* [62–64], with moderate-to-severe depression (e.g., *eCare for Moods*) [65–67], or with severe mental illness (e.g., *Wellness Recovery Action Planning (WRAP*)) [68]. On the other hand, other self-management programs (e.g., the web-based *Big White Wall*) for persons with mild-to-moderate depression [69, 70] or for treatment-resistant moderate-to-severe depression (e.g., *Self-management for Chronic Anxiety and Depression (SemCAD)*) [71] failed to improve depression severity compared to the control group. The SemCAD program did, however, improve self-empowerment in patients with treatment-resistant depression [71]. All the aforementioned studies (except for Zoun et al., [71]) differed from our study in terms of settings, depression severity, and treatment resistance.

### Study implications and future research

Although PPEP4All-PDD did not improve clinical outcomes compared to CAU, patients rated the program with an acceptable score of 6.6, and the majority of patients (78%) recommended the program. Comparing it to the original PPEP4All program, this rating and percentage was lower than the score of 7.9 (*n* = 29) given by patients with manifested Huntington’s disease [27], and the 84% (*n* = 55) of patients with chronic pituitary disease who recommended PPEP4All [32]. However, treatment needs and expectations of patients with PDD may be different than patients with chronic somatic diseases and comorbid depression. This may also help us understand why we did not find an improvement in depressive symptoms, which was one central result in a PPEP4All study with patients with chronic somatic illness and comorbid depressive symptoms [28]. Regardless, these results indicate that there is room for improvement: PPEP4All-PDD can be optimized for patients with treatment-resistant PDD (see our qualitative results in Supplementary Material). In particular, we would like to highlight four important considerations that hold particular implications for self-management programs such as PPEP4All-PDD.

First, PPEP4All-PDD was offered as psychiatric rehabilitation at the end of the treatment process in specialized mental healthcare for patients with treatment-resistant PDD. There was an option to end treatment after completion of PPEP4All-PDD, based on shared decision-making. However, both patients and therapists found it difficult to accept PPEP4All-PDD as the “final” treatment; most patients returned to their usual care therapist after PPEP4All-PDD. Previous research confirms that the process of ending treatment after a certain period is not certain nor clear, even if self-efficacy/empowerment improves [71]. Also, the culture in specialized mental healthcare may not be accustomed to moving from traditional symptom-reduction approaches (e.g., cognitive behavioral therapy) to recovery-focused techniques [67]. Future research can examine the efficacy of offering the program earlier in the treatment process as an empowerment/autonomy-focused program, before patients become demotivated and less hopeful after several unsuccessful treatments.

Second, our finding that depressive symptoms did not improve, relative to CAU, only confirms that the focus of treatment for patients with treatment-resistant PDD should move from symptom reduction to functional recovery. To this point, researchers may need to examine which functional recovery outcomes are amenable to change in patients with treatment-resistant PDD. Improvements in functional ability may be reflected in other outcomes such as empowerment and active coping, for instance, rather than outcomes such as mental resilience and need for care. The original PPEP4All program has shown improved self-efficacy and active coping in previous studies in patients with chronic somatic illness [27, 32].

Third, studies that included patients with chronic/recurrent depression demonstrated that self-management programs may need to be longer (spread across at least 12 months) to be clinically effective [65, 67], and they could be offered (partly) online [63, 65, 72, 73], which could encourage self-monitoring and autonomy [65]. Indeed, the persistence of depression symptoms over time [15] may require an intensive, longer-term program. Patients may require more time to implement self-management tools into their daily lives before this is reflected in an improvement of psychosocial burden or functional recovery. Regarding the online program, there is now a therapist-supported online e-Health version of PPEP4All [74]. Future research could examine whether offering PPEP4All-PDD (in-person or online) with separate homework-discussion sessions and more time between sessions (i.e., offered every 2–3 weeks) would result in improved clinical effectiveness.

Fourth, we did not find support for less psychosocial disease burden in partners/caregivers after PPEP4All-PDD. Based on the qualitative findings, partners/caregivers reported having some prior knowledge regarding caregiver issues and depression, and our quantitative data showed only very mild psychosocial burden prior to PPEP4All-PDD for partners/caregivers, creating a floor effect, which may explain our results. It is important to note that the original PPEP4All protocol advises a minimum of 6 out of 8 sessions for clinical effectiveness [28, 30]. However, we could not confirm this finding in our study. Although we allowed a minimum attendance of 3 sessions, the majority of PPEP4All-partners completed at least 7 out of 9 sessions (11 out of 14; 78.4%). Partners/caregivers of patients with PDD may be different than those of partners/caregivers of patients with other chronic somatic illness, perhaps due to the level of prior knowledge regarding the disease and long-term experience with the patient’s depression. In the future, partners/caregivers suggested that PPEP4All-PDD may be best suited for partners/caregivers who require additional knowledge and support regarding caregiver issues. Considering that we could not compare results with CAU partners/caregivers, the impact on clinical effectiveness for the partner/caregiver in PPEP4All-PDD should be further investigated.

### Strengths and limitations

This study furthers our knowledge regarding the clinical effectiveness of psychiatric rehabilitation/self-management for the complex population of patients with treatment-resistant PDD and their partners/caregivers. In our mixed-methods pragmatic RCT, we collected both quantitative and qualitative data from various perspectives (PPEP4All-PDD/CAU patients, PPEP4All-PDD partners/caregivers, and PPEP4All-PDD therapists). In addition, treatment was provided in a naturalistic setting, reflecting day-to-day clinical practice. This increases external validity, thereby enhancing the generalizability of the results to clinical practice. Moreover, our study has good internal validity as is demonstrated by the successful randomization of the treatment groups and the high treatment adherence.

This study also has its limitations. First, the sample size was smaller than we intended, and we had high attrition at the final assessment (loss of 50% of participants). Our sample size was calculated based on cost-effectiveness, for which we aimed to include a total sample of 178 participants with PDD [35]. Especially in the time of the COVID-19 pandemic, there were some critical challenges in terms of recruitment of this population, who, because past unsuccessful treatment, may have become hopeless, passive, and frustrated. Due to the risk of randomization, which could result in disappointment/further frustration, some patients refused research participation, and therapists assessed whether treatment of patients at the end of their treatment process could be ended without research participation. Patients may also have refused participation due to fear of ending treatment. This resulted in a smaller eligible sample pool, leaving us with patients who had either milder depressive symptoms (and shorter treatment periods) or more severe depressive symptoms (with greater avoidance and anxiety). If CAU participants dropped out or completed our study, they could receive PPEP4All-PDD without further research participation if they so desired.

Second, it is not clear how the COVID-19 pandemic impacted the results of our study. The pandemic may have negatively affected mental health [75], resulting in poorer clinical outcomes regardless of treatment. Patients often continued usual treatment after completion of PPEP4All-PDD, thus limiting the PPEP4All-PDD program as a psychiatric rehabilitation program. Additionally, a change in the (administration of) standard care in mental health clinics during the pandemic may have also affected our trial, possibly reducing generalizability to the non-pandemic situation. We expected CAU patients to receive mainly supportive non-protocolized/unspecific sessions; the content of CAU was only partly confirmed in our evaluation survey. The COVID-19 situation may have played a major role in this finding, as patients may have needed more care than usual, making it difficult to generalize results to normal treatment procedures prior to COVID-19. Finally, due to COVID-19 prevention regulations, we offered PPEP4All-PDD sessions via online video calls. A few patients mentioned that they would have preferred face-to-face sessions, however, possibly due to the experience of loneliness [76].

Third, although we found high treatment protocol adherence in PPEP4All-PDD therapists (> 85%), this was only measured with a checklist, which was completed by the PPEP4All-PDD therapist. Audio or video recordings of the treatment sessions were not logistically possible. Therefore, while PPEP4All-PDD therapists confirmed they provided the principal components of PPEP4All-PDD, there may have been some differences in the delivery of the program.

Fourth, more PPEP4All-PDD than CAU participants changed medication during our study. Explorative mixed-model analyses, adjusted for medication changes, did not change our previous non-adjusted results. However, a limitation of these analyses is that each time point was adjusted, as we did not have data regarding the specific date of the medication change. We could only confirm that the change did not take place during PPEP4All-PDD.

## Conclusion

This study furthers our clinical knowledge regarding psychiatric rehabilitation through self-management interventions (such as PPEP4All-PDD) for a complex population of patients with treatment-resistant PDD and their partners/caregivers. Contrary to our hypotheses, we found no evidence for superiority of PPEP4All-PDD over CAU in terms of clinical effectiveness. There was also no significant improvement in psychosocial burden for PPEP4All-PDD partners/caregivers after completion of the program. Although 78% of patients recommended PPEP4All-PDD, there was no significant difference in treatment satisfaction between PPEP4All-PDD and CAU. Further clinical research on the PPEP4All-PDD program is recommended, after further amending the program for patients with PDD based on our qualitative suggestions. Potential improvements include offering PPEP4All-PDD as a longer-term or online program, possibly earlier in the treatment process.

### Supplementary Information

**Additional file 1.** Supplementary material.

## Data Availability

Datasets generated and/or analyzed during the current study will be pseudonymized and stored on an online Dutch meta-data catalogue called the Data Archiving and networked Services (DANS, www.dans.knaw.nl), according to the funding sponsor policy, with access limited to a designated team within the Department of Psychiatry of the Leiden University Medical Center. External researchers may get access to the final trial dataset from the designated team on reasonable request. The (intellectual) property rights with regard to the generated data will reside at the Leiden University Medical Center. Anonymized results will be published in peer-reviewed journals and presented in international conferences.
