# Supplementary material for: The clinical effectiveness of a self-management intervention for patients with persistent depressive disorder and their partners/caregivers: results from a multicenter, pragmatic randomized controlled trial
Source: Trials. 2024 Mar 13;25:187. doi: 10.1186/s13063-024-08033-9 (PMC10938802; doi:10.1186/s13063-024-08033-9)
Supplement: Supplementary file 1 — Additional file 1. Supplementary material. [file 13063_2024_8033_MOESM1_ESM.docx]

**SUPPLEMENTARY MATERIAL.**

**Table 1.** Overview of study outcome measures in the PPEP4All-PDD and CAU conditions by measurement time point for patients and partners/caregivers.

| **Measures** | **0 mo.**  **(T1)** | **3 mo.**  **(T2)** | **6 mo.**  **(T3)** | **12 mo.**  **(T4)** | **S1** | **S2** |
| --- | --- | --- | --- | --- | --- | --- |
| Demographics* | X |  |  |  | X | X |
| Clinical depression/suicidality screening (MINI)^a^ | X |  |  |  | - | - |
| Quality of life (EQ-5D-5L)* | X | X | X | X | X |  |
| Healthcare costs (TiC-P) | X | X | X | X | X |  |
| (Residual) depressive symptoms (IDS-SR) | X | X | X | X |  | X |
| Psychopathology (SQ-48) | X | X | X | X |  | X |
| Happiness (SRH) | X | X | X | X |  | X |
| Mental resilience (BRSnl) | X | X | X | X |  | X |
| Burden of living with chronic disease (B4CZ) | X | X | X | X |  | X |
| Burden living with chronic disease (B4CZ-Partner)* | X | X | X | X |  | X |
| Evaluation survey^b^* |  |  |  | X |  | X |

*Note*. PPEP4All-PDD and CAU patients were invited to complete all measures except the B4CZ-Partner, unless the participant dropped out early. S1 refers to main measures used in Study 1 regarding the cost-effectiveness of PPEP4All-PDD (although additional measures were used for the purposes of imputation of missing values). S2 refers to the main measures used Study 2 regarding the clinical effectiveness of PPEP4All-PDD.

*Abbreviations.* S1 = Study 1. S2 = Study 2. Mo. = months. MINI = Mini-International Neuropsychiatric Interview. EQ-5D-5L = EuroQoL-5 with visual analogue scale. TiC-P = Trimbos Medical Technology Assessment questionnaire for psychiatric illness-associated costs. IDS-SR = Inventory of Depressive Symptomatology, self-rated. SQ-48 = Symptom Questionnaire-48. SRH = self-rated happiness. BRSnl = Brief Resilience Scale. B4CZ(-Partners) = Questionnaire on Burden of Chronic Disease for Patients (or Partners).

^a^ Modules A, B, C regarding (chronic) depression, dysthymia, and suicidality were used.

^b^ Survey included questions regarding medication changes, treatment received, treatment satisfaction, and recommendation of PPEP4All-PDD.
* denotes the questionnaire that are administered to PPEP4All-PDD partners/caregivers in the study; no CAU partners/caregivers were included in the study.

**Table 2.** Topic list for PPEP4All-PDD patients, - partners/caregivers, - therapists: Satisfaction with PPEP4All-PDD (see also Chambers et al., 2015; Kok and van Wijngaarden, 2003).

| **Interview Question** |
| --- |
| You received PPEP4All. How was your experience with PPEP4All?  *Follow-up:* what did you think of it?  PPEP4All therapist variation: You provided PPEP4All. How was your experience with PPEP4All as therapist? * |
| What effect/result did PPEP4All have on you? |
| What did you learn from PPEP4All? |
| After PPEP4All, did you make any changes in your life?  *Follow-up:* Would your life be different if you did not receive PPEP4All? |
| What did you think about your PPEP4All therapist? |
| What was positive about PPEP4All? * |
| What was negative, or less positive, about PPEP4All? * |
| What score would you give PPEP4All? [where 1=horrible/very bad to 10=outstanding] * *Follow-up:* Could you further explain your score? |
| Do you have any comments and/or suggestions to improve PPEP4All? * |

*Notes.* For PPEP4All-PDD therapists: questions with an asterisk* were asked.


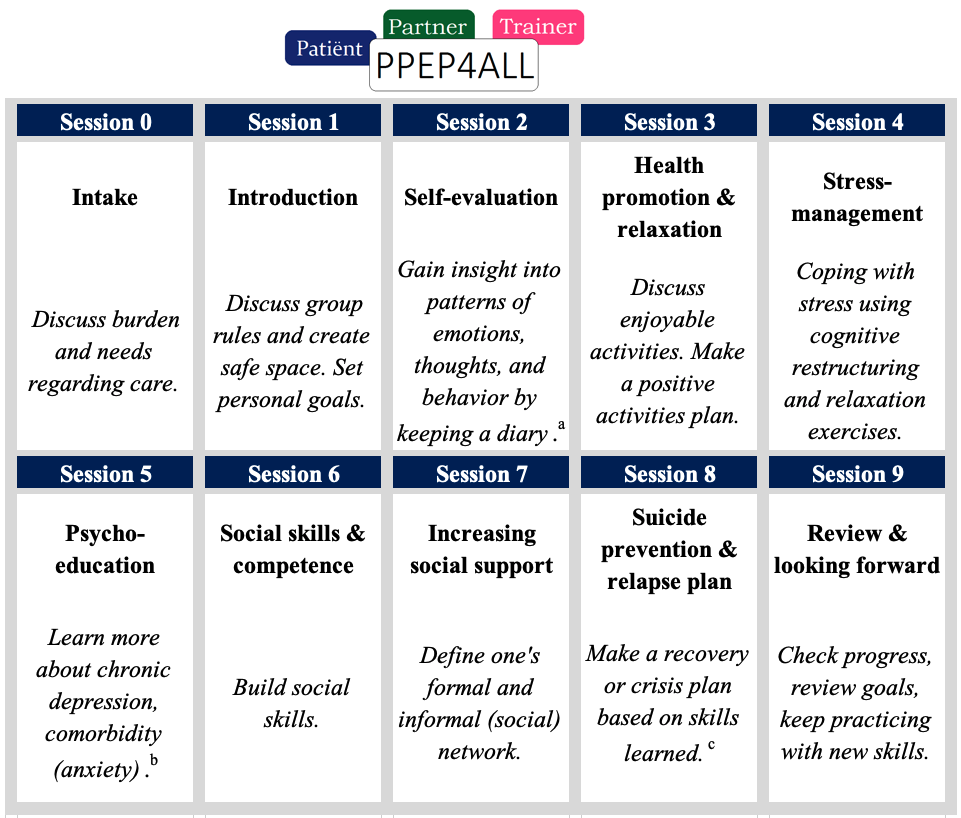
**Figure A.** An overview of the adapted PPEP4All-PDD self-management themes per session for patients with PDD and their partners/caregivers.

*Note.* PPEP4All-PDD = Patient and Partner Education Program for All Chronic Diseases-Persistent Depressive Disorder.
In relation to the generic/general PPEP4All, the following adaptations for PDD were made: session 5 included in-depth psychoeducation regarding PDD, and session 8 was added to provide essential information regarding suicide/relapse prevention. Each session has a general structure of discussing previous homework, presenting active information (i.e., the theme), performing an active exercise, then providing homework and a preview of the next session. Moreover, patient and partner/caregiver programs generally include the same themes, with further specifications for session 5 and 8.

^a^ Participants also gain insight by using a body awareness exercise.

^b^ In partner/caregiver program, session 5 focuses on caregiver burden and stress (overload) and relevant challenges.

^c^ In partner/caregiver program, session 8 focuses on making a self-care plan.

**Table 3.** *Adjusted mean differences between conditions in clinical effectiveness outcomes at each time assessment using mixed-model analyses.*

| **Outcome** | **Time in months (Assessment)** | **CAU**  **Adjusted mean (95% CI)** | **PEPP4All-PDD**  **Adjusted mean (95% CI)** | **Δ CAU - PPEP4All-PDD**  **Adjusted means (95% CI)** | ***p*-value** |
| --- | --- | --- | --- | --- | --- |
| IDS-SR | 0 (T1) | 36.5 (29.8; 43.3) | 35.1 (28.6; 41.6) | 1.44 (-8.3; 11.2) | 0.77 |
| IDS-SR | 3 (T2) | 31.4 (24.7; 38.2) | 32.8 (26.2; 39.5) | -1.38 (-11.2; 8.4) | 0.78 |
| IDS-SR | 6 (T3) | 29.5 (22.4; 36.5) | 30.7 (24.0; 37.5) | -1.27 (-11.4; 8.8) | 0.81 |
| IDS-SR | 12 (T4) | 32.7 (25.4; 40.0) | 30.0 (23.4; 36.5) | 2.75 (-7.3; 12.8) | 0.59 |
| SQ-48 | 0 (T1) | 62.8 (51.9; 73.7) | 66.2 (55.8; 76.7) | -3.44 (-19.1; 12.2) | 0.67 |
| SQ-48 | 3 (T2) | 63.0 (52.1; 73.9) | 62.2 (51.7; 72.8) | 0.74 (-15.0; 16.5) | 0.93 |
| SQ-48 | 6 (T3) | 53.5 (42.3; 64.7) | 59.3 (48.5; 70.0) | -5.76 (-21.9; 10.4) | 0.48 |
| SQ-48 | 12 (T4) | 60.4 (48.9; 71.9) | 56.5 (46.1; 67.0) | 3.85 (-12.2; 19.9) | 0.64 |
| SRH | 0 (T1) | 4.7 (4.1; 5.4) | 4.7 (4.1; 5.3) | 0.05 (-0.9; 1.0) | 0.92 |
| SRH | 3 (T2) | 4.5 (3.8; 5.1) | 4.4 (3.8; 5.0) | 0.06 (-0.9; 1.0) | 0.91 |
| SRH | 6 (T3) | 4.5 (3.8; 5.1) | 4.1 (3.5; 4.8) | 0.34 (-0.6; 1.3) | 0.48 |
| SRH | 12 (T4) | 4.4 (3.7; 5.1) | 4.3 (3.7; 4.9) | 0.17 (-0.8; 1.1) | 0.73 |
| B4CZ: Bb | 0 (T1) | 24.9 (17.8; 31.9) | 29.6 (22.9; 36.4) | -4.75 (-14.8; 5.3) | 0.36 |
| B4CZ: Bb | 3 (T2) | 23.0 (16.0; 30.0) | 25.9 (19.1; 32.7) | -2.86 (-13.0; 7.3) | 0.58 |
| B4CZ: Bb | 6 (T3) | 19.0 (11.8; 26.3) | 21.7 (14.8; 28.7) | -2.72 (-13.2; 7.7) | 0.61 |
| B4CZ: Bb | 12 (T4) | 20.8 (13.3; 28.3) | 22.5 (15.7; 29.2) | -1.68 (-12.1; 8.7) | 0.75 |
| B4CZ: NfH | 0 (T1) | 28.6 (21.4; 35.9) | 30.4 (23.5; 37.4) | -1.78 (-12.2; 8.6) | 0.74 |
| B4CZ: NfH | 3 (T2) | 29.7 (22.5; 37.0) | 27.2 (20.1; 34.2) | 2.60 (-7.8; 13.0) | 0.63 |
| B4CZ: NfH | 6 (T3) | 22.9 (15.4; 30.5) | 25.4 (18.1; 32.7) | -2.47 (-13.3; 8.4) | 0.66 |
| B4CZ: NfH | 12 (T4) | 23.2 (15.3; 31.1) | 23.2 (16.2; 30.2) | -0.01 (-10.8; 10.8) | 1.00 |
| BRS | 0 (T1) | 2.1 (1.8; 2.4) | 2.2 (1.9; 2.5) | -0.08 (-0.5; 0.4) | 0.73 |
| BRS | 3 (T2) | 2.4 (2.1; 2.8) | 2.3 (2.0; 2.6) | 0.13 (-0.3; 0.6) | 0.57 |
| BRS | 6 (T3) | 2.3 (2.0; 2.6) | 2.4 (2.1; 2.7) | -0.11 (-0.6; 0.4) | 0.66 |
| BRS | 12 (T4) | 2.3 (2.0; 2.7) | 2.4 (2.1; 2.7) | -0.08 (-0.6; 0.4) | 0.75 |

*Abbreviations.* PPEP4All-PDD = Patient and Partner Education Program for All Chronic Diseases-Persistent Depressive Disorder; CAU = Care as Usual; CI = Confidence Interval; IDS-SR = Inventory of Depressive Symptomatology-Self Report; SQ-48 = Symptom-Questionnaire 48; SRH = Self-Rated Happiness; B4CZ: Bb = Questionnaire on Burden of Chronic Disease for Patients – Bothered-by Problems; B4CZ: NfH = Questionnaire on Burden of Chronic Disease for Patients – Need for Help; BRS = Brief Resilience Scale.


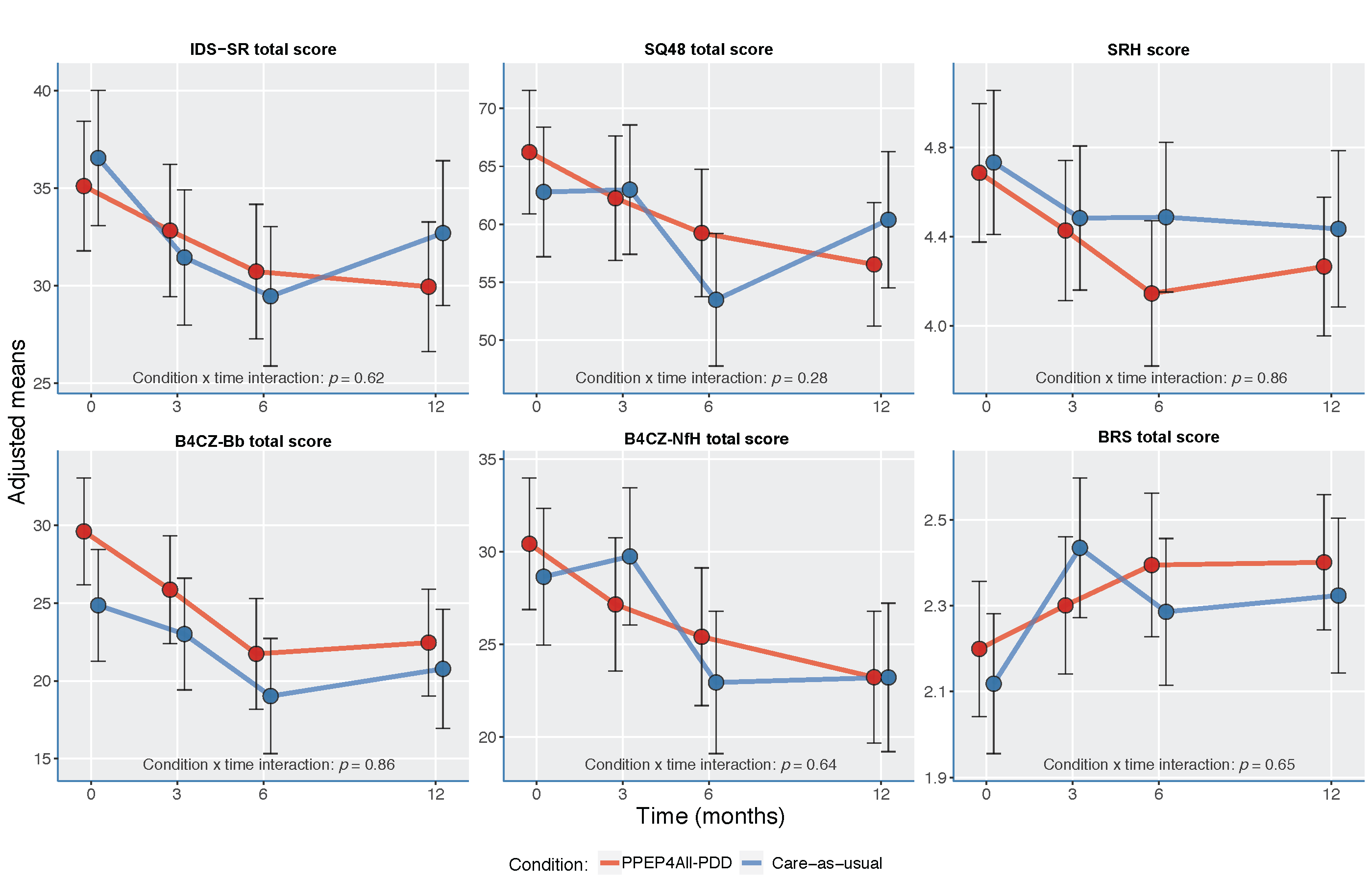
**Figure B***. Explorative mixed model analyses for the clinical effectiveness outcomes at each time assessment, adjusted for medication changes, as reported on the general evaluation survey.*

**Table 4.** Qualitative treatment feedback from PPEP4All-PDD patients, - partners/caregivers, - therapists.

| **Treatment Feedback** (*with example opinion of research participant in italics*) | **Patients**  **(*n* = 24)** | **Caregivers**  **(*n* = 9)** | **Therapists**  **(*n* = 10)** |
| --- | --- | --- | --- |
| ***COMPLIMENTS*** | | | |
| Group/individual format (specific advantages/choice between formats)  *Example opinion “It was nice to experience support and know that you aren’t the only one.”[patient]* | 8 | 6 | 3 |
| Skills learned  *Example opinion: “I learned to* *recognize signals and personal pitfalls and to apply strategies. I learned tools, and I feel so much better now.” [patient]* | 7 | 2 | 0 |
| Workbook availability/structure  *Example opinion: “Nice, clear structure and useful to work with the workbook.” [partner]* | 3 | 3 | 4 |
| Program goal/organization/themes  *Example opinion: “[PPEP4All-PDD] gives the practitioner the opportunity to lean back and give the responsibility to the patient. Self-management is central, which makes PPEP4All sessions less stressful and makes it inviting to participate. The method itself is clear; it provides structure, with no loss of treatment focus. [PPEP4All-PDD] is also visually strong. Each module has the same structure; good design.” [therapist]* | 9 | 6 | 10 |
| Caregiver involvement in patient treatment was appreciated  *Example opinion: “It was great that partners are given attention; I gained more insight that I also matter and that I do not need to ignore my needs.” [partner]* | 0 | 2 | 1 |
| Therapist (e.g. friendly/sympathetic, knowledgeable, listened well; only feedback from patients and partners)  *Example opinion: “The therapist (group leader) did an excellent job and listened to everyone and gave everyone their turn [to speak].”[patient]* | 8 | 6 | 0 |
| Patients’ positive posttreatment changes (psychosocial/motivational/communication; only feedback from patients and partners)  *Example opinion:* *“It brought about many changes for my wife and therefore also for myself.” [partner]* | 6 | 2 | 2 |
| ***SUGGESTIONS FOR IMPROVEMENT*** | | | |
| Make sessions biweekly (instead of weekly)  *Example opinion: “… I needed more time to process things. It was exhausting.” [patient]* | 2 | 0 | 0 |
| Program extension (longer duration) or a follow-up session  *Example opinion: (1)“The program was too short for me to view it as therapy.”[patient]*  *(2) “There was no follow-up, and I felt like I needed an extra consultation from the psychologist. After-care is desirable.” [patient]*  *(3)“[I completed a different group before], and what is very nice about that, although it is much more of an intensive course… you have a number of modules that you have to work through. And then one day you get the theory and then you have a few days of homework time, and then another day is completely [to discuss] homework. So you have the time to let each other talk during the session. But it was not like that [with PPEP4All-PDD].” [patient]* | 5 | 2 | 1 |
| Set clearer rules regarding absences  *Example opinion: “I feel like I did not fully get everything from the program because some group participants were absent. There was insufficient discipline from other participants to be present at each session. There needs to be stricter rules set for participants.” [patient]* | 6 | 3 | 0 |
| Clarifying partner/caregiver's expectations or patient motivation/treatment history prior to program  *Example opinion: “[At first] I didn’t understand some parts at all…And at some point it got a bit better, and then I started to like it more and more… It is important to discuss expectations in advance. Why do you want to do it? Do you know that some people have an expectation? …And you could also say in advance, don’t go into [PPEP4All-PDD] thinking that you will get pre-chewed and ready-to-eat chunks, or however you want to say it. I think it’s important for prospective [participants] to think about … PPEP4All-PDD, that they don’t have the wrong expectations about it.” [partner]* | 3 | 1 | 3 |
| Therapist providing more examples to clarify workbook/homework  *Example opinion: “I would have liked more examples from one’s one experiences, instead of just providing the ones in the workbook. We can learn a lot from that.”[patient]* | 1 | 1 | 0 |
| Revise workbook (shorter, more PDD-specific)  *Example opinion: “It is particularly suitable for people who are not yet well-acquainted with depression. Provided it is given in normal/easy-to-understand language…”[patient]* | 4 | 4 | 4 |
| Implement digital app for homework/self-evaluation  *Example opinion: (1) “Sometimes the program is very extensive/broad; I would suggest using an online app to aid self-evaluation.”[therapist]*  *(2) “I would prefer to use a website that you could use to save your work.”[patient]* | 1 | 0 | 1 |
| Allow two therapists to give group program (instead of one)  *Example opinion: “We enjoyed giving the program together and to fill in the gaps for each other.” [therapist]* | 0 | 0 | 1 |
| Ensure the maximum group size (max. 6) (to allow sufficient time per person)  *Example opinion: (1)“Sometimes you need 10 minutes extra to discuss a section. I think it is unfortunate to say, “oh, now we need to do this [instead].” You would want to continue [with the section], but sometimes you can’t. The group can’t be too big, because otherwise you don’t have enough time to give every person attention.” [therapist]*  *(2)“It went too fast for me. It felt like a school day and I needed to wait for my turn. I wanted to be able to tell more about myself.”[patient]* | 3 | 0 | 3 |
| Offer program (also) earlier in the depression treatment process (i.e. also for non-chronic depression patients)  *Example opinion: (1)“It is good to know who participates in the program. I think if people are currently undergoing a severe/deep depression, then we need to evaluate whether this is the right time for them to participate.”[therapist] (2) “I think that it is important to look closely at the motivation of the patient and also to look at their treatment history. If someone is already receiving therapy for a long time, what will [PPEP4All-PDD] contribute?” [therapist]* | 3 | 1 | 4 |

*Note.* This table is based on the evaluation survey and/or the qualitative individual interview of PPEP4All-PDD research participants (patients/caregivers/therapists) who were willing to give their personal opinion on the PPEP4All-PDD program.

*Abbreviations.* PPEP4All-PDD = Patient and Partner Education Program for All Chronic Diseases-Persistent Depressive Disorder.
